# Efficient genome editing of wild strawberry genes, vector development and validation

**DOI:** 10.1111/pbi.12922

**Published:** 2018-04-24

**Authors:** Junhui Zhou, Guoming Wang, Zhongchi Liu

**Affiliations:** ^1^ Department of Cell Biology and Molecular Genetics University of Maryland College Park MD USA; ^2^ State Key Laboratory of Crop Genetics and Germplasm Enhancement Centre of Pear Engineering Technology Research Nanjing Agricultural University Nanjing Jiangsu China

**Keywords:** strawberry, CRISPR, vector development, high efficiency, auxin, inheritance

## Abstract

The clustered regularly interspaced short palindromic repeats (CRISPR)–Cas9 system is an effective genome editing tool for plant and animal genomes. However, there are still few reports on the successful application of CRISPR–Cas9 to horticultural plants, especially with regard to germ‐line transmission of targeted mutations. Here, we report high‐efficiency genome editing in the wild strawberry *Fragaria vesca* and its successful application to mutate the auxin biosynthesis gene *TAA1* and *auxin response factor 8* (*ARF8*). In our CRISPR system, the *Arabidopsis* U6 promoter AtU6‐26 and the wild strawberry U6 promoter FveU6‐2 were each used to drive the expression of sgRNA, and both promoters were shown to lead to high‐efficiency genome editing in strawberry. To test germ‐line transmission of the edited mutations and new mutations induced in the next generation, the progeny of the primary (T0) transgenic plants carrying the CRISPR construct was analysed. New mutations were detected in the progeny plants at a high efficiency, including large deletions between the two PAM sites. Further, T1 plants harbouring *arf8* homozygous knockout mutations grew considerably faster than wild‐type plants. The results indicate that our CRISPR vectors can be used to edit the wild strawberry genome at a high efficiency and that both sgRNA design and appropriate U6 promoters contribute to the success of genomic editing. Our results open up exciting opportunities for engineering strawberry and related horticultural crops to improve traits of economic importance.

## Introduction

*Fragaria vesca* (*F. vesca*), the diploid wild strawberry, is an important member of the *Rosaceae* family and an emerging model system for the cultivated strawberry (*Fragaria × ananassa*) as well as other *Rosaceae* species. *F. vesca* has a small and sequenced genome (2*n* = 14, 240 Mb genome), relatively short life cycle, ease of growth and, most importantly, facile transformation (Shulaev *et al*., 2011). While there have been extensive transcriptome data in *F. vesca* informing the expression pattern of genes during development (Hawkins *et al*., 2017; Hollender *et al*., 2014; Kang *et al*., 2013), the function of most genes is not determined due to a lack of efficient gene knockout method. Establishing an efficient genome editing technology for *F. vesca* will provide an incredibly useful tool for studying gene function and add another resource to this emerging model. Further, it holds the promise for manipulating genes in cultivated strawberry to improve traits of economic importance.

RNA‐guided nucleases from the clustered regularly interspaced short palindromic repeat (CRISPR) are a powerful genome editing technology due to their high efficiency, ease of manipulation and high specificity (Ding *et al*., 2016; van der Oost, 2013; Voytas, 2013). CRISPR/Cas9 employs the endonuclease activity of Cas9 to produce double‐strand breaks (DSBs) at target genomic sites, with the specificity of the cutting site determined by an engineered single guide RNA (sgRNA). DSBs caused by the sgRNA/Cas9 complex initiate the DNA repair in the host cells, including the nonhomologous end‐joining (NHEJ) pathway, which is error‐prone and often introduces small deletions or insertions (Cong *et al*., 2013; Hsu *et al*., 2014; Nekrasov *et al*., 2013; Voytas, 2013). The CRISPR/Cas9 system has been widely applied both to animal and plant research (Cong *et al*., 2013; Hsu *et al*., 2014; Jinek *et al*., 2013; Mali *et al*., 2013). To date, successful genome editing has been reported in rice (Shan *et al*., 2013), *Arabidopsis* (Mao *et al*., 2013), tobacco (Nekrasov *et al*., 2013), tomato (Brooks *et al*., 2014), maize (Xing *et al*., 2014), wheat (Wang *et al*., 2014), potato (Wang *et al*., 2015a), barley (Lawrenson *et al*., 2015), Brassica (Lawrenson *et al*., 2015) and others, offering an unprecedented opportunity to conduct functional genomics in crop plants.

The *Rosaceae* is an economically important plant family that includes many important fruit crops such as apple, pear, peach, plum, raspberry and strawberry. There have been limited reports of successful CRISPR/Cas9 genome editing in *Rosaceae* plants. In apple, CRISPR/Cas9 genome editing was reported to occur in apple protoplasts (Malnoy *et al*., 2016) and in callus‐generated shoots (Nishitani *et al*., 2016). However, there has been no report of successful inheritance of CRISPR‐induced mutations as most of the mutations were generated in somatic cells that could not yield progeny. Additionally, most of these studies targeted the phytoene desaturase (PDS) gene, whose mutations led to the death of the shoots. Therefore, applying CRISPR/Cas9 to editing nonmarker genes, testing the germ‐line inheritance of the CRISPR/Cas9‐induced mutations and evaluating Cas9 activity in subsequent generations will help determine the feasibility of applying the CRISPR/Cas9 technology to improving *Rosaceae* fruit crops. Further, there has been no report of any CRISPR/Cas9 studies in strawberry.

In this study, we reported the successful genome editing of wild strawberry *Fragaria vesca*, targeting an auxin biosynthesis gene *TAA1* and an auxin response factor *ARF8* in T0 and T1 generations. The mutation rate ranged from 49% to 75% in the T0 generation and was higher in the T1 generation. Different types of mutations were generated including nucleotide deletion and substitution. Interestingly, large fragment deletions between the two sgRNA target sites within the same gene were observed in the T1 generation. The vectors created in this study and the demonstrated high efficiency in the wild strawberry add an essential tool to strawberry research and help establish *F. vesca* as an excellent model for dissecting gene function.

## Results

### Construct CRISPR genome editing vectors for *Fragaria vesca*

**Figure 1:**
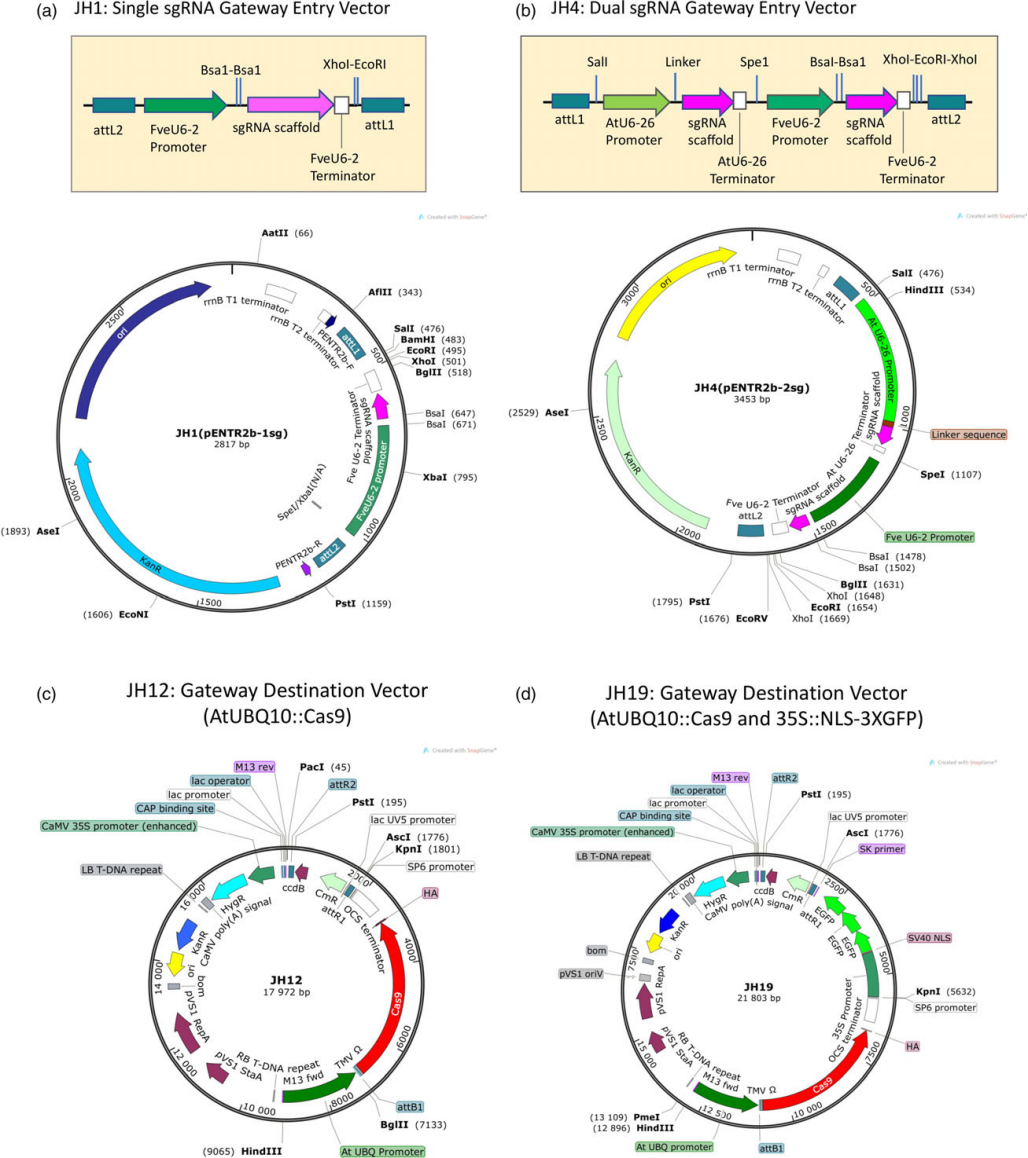
Diagrams illustrating gateway‐based CRISPR vectors. (a) Illustration of the single sgRNA gateway entry vector JH1. A sgRNA cloning cassette (enlarged on top) is inserted between the attL1 and attL2 recombination sites of pENTR2b. Two inverted BsaI restriction enzyme recognition sites enable easy cloning of the double‐stranded target sequence. pENTR2b‐R and pENTR2b‐F in the vector map are two sequencing primers. (b) Illustration of the dual sgRNA gateway entry vector JH4. The dual sgRNA cloning cassette (enlarged on top) is inserted between the attL1 and attL2 recombination sites of pENTR2b. One sgRNA is driven by the FveU6‐2 promoter similarly to JH1. The second sgRNA is driven by the *Arabidopsis* U6‐26 promoter. A linker sequence GCGCTTCAAGGTGCACATGG between the AtU6‐26 promoter and the sgRNA scaffold can be replaced with a 20 bp target sequence using the Q5 site‐directed mutagenesis kit. (c) The gateway destination vector JH12 is a hygromycin‐resistant binary vector harbouring a AtUBQ10 promoter‐driven Cas9. (d) The gateway destination vector JH19, a binary vector similar to JH12 except that JH19, harbours a 35S::NSL‐3XGFP cassette. The GFP fluorescence allows easy visualization and identification of transgenic plants.

To construct a CRISPR genome editing vector that would work in strawberry with a high efficiency, we made a construct (named JH1), where the single sgRNA was driven by the *Fragaria vesca* U6‐2 promoter (Figure 1a). To insert a seed RNA with homology to the target sequence, synthesized forward‐strand and reverse‐strand oligonucleotides were annealed and then ligated to the vector that was linearized with the BsaI type II restriction enzyme. Second, a dual sgRNA entry vector (JH4) was constructed (Figure 1b), where one seed RNA was inserted at the BsaI site driven by the *FveU6‐2* promoter just as described for JH1 and a second seed RNA was inserted into the JH4 vector via the Q5 site‐directed mutagenesis kit. This second sgRNA was driven by the *Arabidopsis* U6‐26 promoter. The pENTR vector JH4 can also be stacked to insert 4, 6, 8 or a higher number of sgRNAs into the pENTR vector (Figure S1).

The above pENTR vectors, JH1 and JH4, can be easily recombined via gateway technology into various destination vectors that harbour the *Cas9* gene. Destination vector JH12, a hygromycin‐resistant binary vector, was constructed (Figure 1c). This vector harbours a UBQ promoter‐driven Cas9, which was codon‐optimized for *Arabidopsis* and maize (Peterson *et al*., 2016). A second destination vector JH19 (Figure 1d) is similar to JH12 except that it contains a 35S::NSL‐3XGFP cassette allowing easy visualization and identification of transformants and transgenic seedlings (Figure S2). This feature enables tracking of the CRISPR vector in future generations. Additional destination vectors include JH17 and JH16. In JH17, the *FveYAO* (gene28947) promoter from *F. vesca* was used to drive *Cas9* (Figure S3). In JH16, an egg‐cell‐specific *FveECL1* (gene02682) promoter from *F. vesca* was used to drive *Cas9* (Figure S3). The *YAO* promoter is expressed in actively dividing cells and may enhance *Cas9* expression in callus during transformation. The *ECL1* promoter may facilitate germ‐line genome editing if one applies floral dipping method to strawberry transformation.

### Test genome editing efficiency in *Arabidopsis* protoplasts

As strawberry germ‐line transformation takes 9 months, a transient system was first used to test the efficiency of the constructed CRISPR vectors. A mutated GFP with a frameshift mutation was previously used as a readout for CRISPR genome editing in a tobacco transient expression system (Jiang *et al*., 2013). A seed RNA targeting the GFP frameshift mutation site may induce insertion or deletion mutations, correcting (at about 33% chance) the reading frame and restoring the GFP (Jiang *et al*., 2013).

The 35S::GFPm was cotransfected into *Arabidopsis* protoplasts with JH12 harbouring Cas9 and sgRNA targeting the GFPm. The efficiency between *FveU6* promoter‐driven sgRNA and *AtU6* promoter‐driven sgRNA (both in the JH12 vector) was compared. Although both sgRNAs resulted in GFP‐positive protoplasts indicating successful genome editing, a higher editing efficiency was observed for the *AtU6*‐driven sgRNA (Figure S4), which may merely indicate preference of the *AtU6* promoter in protoplasts derived from the same species.

### High genome editing efficiencies at the targeted sites in *F. vesca* plants

**Figure 2:**
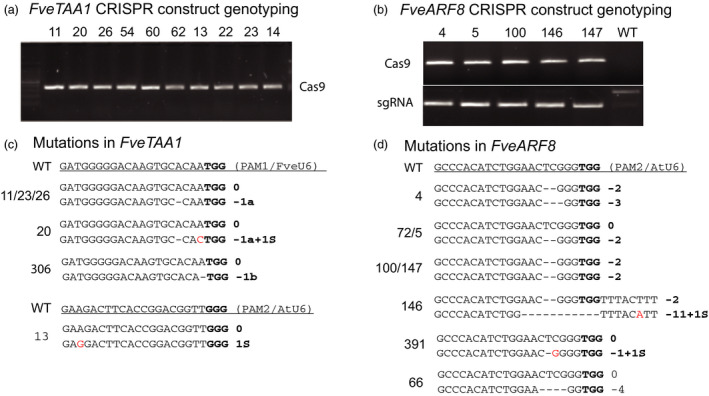
Analyses of T0 wild strawberry plants carrying CRISPR constructs revealed mutations in *FveTAA1* and *FveARF8* genes. (a) PCR genotyping results of T0 transgenic plants harbouring the *JH19‐FveTAA1* construct. The presence of Cas9 PCR product is shown. (b) PCR genotyping results of T0 transgenic plants harbouring the *JH19‐FveARF8* construct. The presence of Cas9 (top row) and sgRNA (lower row) in these transgenic plants is shown by the PCR bands. (c) Sequencing of PCR products revealed the presence of mutations in the *FveTAA1* gene, more abundantly around the PAM1 (TGG) site. Individual transgenic plants are named with a number shown to the left. 11/23/26 refer to three different plants having the same genotype. All mutations exist as heterozygous. (d) Sequencing of PCR products revealed the presence of mutations in the *FveARF8* gene at the PAM2 (TGG) site only. Individual plant names (numbers) are to the left of the sequence. Mutations exist as heterozygous, homozygous or biallelic.

Auxin is known to play important roles in plant growth and development. We designed seed RNAs targeting the auxin biosynthesis gene *FveTAA1* (gene03586) and auxin response factor gene *FveARF8* (gene31631) using the dual sgRNA cassette in the pENTR vector JH4, which was then recombined into JH19 (Figure 1D). For each gene, two seed RNAs were designed to target two different regions of the same gene (Figure S5). *Agrobacterium*‐mediated transformation via tissue culture was performed, and the resulting T0 plants were analysed. First, PCR was used to detect *Cas9* and sgRNA in the T0 transgenic plants to confirm that they were true transgenic plants (Figure 2a,b). Next, the target sequences spanning both PAM1 and PAM2 sites were PCR amplified from the genome and sequenced to detect mutations.

Among the 35 T0 plants harbouring the JH19‐FveTAA1 construct (Figure 2a), 17 possessed mutations (Table 1), indicating a frequency of 49%. Interestingly, half of the mutations were found near PAM1 site and the other half were found near the PAM2 site (Table 1). However, for PAM1 (targeted by the *FveU6‐*driven sgRNA), most mutations were heterozygous (Figure 2c). For PAM2, sequencing results indicated a mixture of multiple mutations in multiple cells. The genetic mosaics caused by different mutations in different cells indicate that the Cas9 may act during later stages of shoot development.

**Table 1:** Summary of genotyping results in T0 transgenic lines

Gene name and plant no.	Line no.	Genotype^a^	PAM site	Confirmation method	Notes
JH19‐**TAA1**‐1	Line 5	(0, −1a)	PAM1(340 bp)	Sequencing	
JH19‐**TAA1**‐3	Line 207	NA	PAM2(470 bp)	Sequencing	
JH19‐**TAA1**‐7	Line 208	NA	PAM2(470 bp)	Sequencing	
JH19‐**TAA1**‐8	Line 208	NA	PAM1(340 bp)	Sequencing	Mismatch starts 240 bp upstream of PAM1
JH19‐**TAA1**‐9	Line 208	NA	PAM2(470 bp)	Sequencing	
JH19‐**TAA1**‐11	Line 5	(0, −1a)	PAM1(340 bp)	Sequencing	Figure 2
JH19‐**TAA1**‐13	Line 17	(0, 1a)	PAM2(470 bp)	Sequencing	Figure 2
JH19‐**TAA1**‐14	Line 209	NA	PAM2(470 bp)	Sequencing	
JH19‐**TAA1**‐20	Line 35	(0, −1a+1S)	PAM1(340 bp)	Sequencing	Figure 2
JH19‐**TAA1**‐22	Line 8	NA	PAM1(340 bp)	Sequencing	
JH19‐**TAA1**‐23	Line 39	(0, −1a)	PAM1(340 bp)	Sequencing	
JH19‐**TAA1**‐25	Line 109	NA	PAM2(470 bp)	Sequencing	
JH19‐**TAA1**‐26	Line 7	(0, −1a)	PAM1(340 bp)	Sequencing	Figure 2
JH19‐**TAA1**‐45	Line 200	NA	PAM2(470 bp)	Sequencing	
JH19‐**TAA1**‐54	Line 5	NA	PAM1(340 bp)	Sequencing	
JH19‐**TAA1**‐60	Line 6	NA	PAM1(340 bp)	Sequencing	
JH19‐**TAA1**‐306	Line 8	(0, −1b)	PAM1(340 bp)	Sequencing	Figure 2
JH19‐**ARF8**‐4	Line 102	(−2, −3)	PAM2(360 bp)	Sequencing	Figure 2
JH19‐**ARF8**‐5	Line 102	(0, −2)	PAM2(360 bp)	Sequencing	Figure 2
JH19‐**ARF8**‐66	Line 15	(0, −4)	PAM2(360 bp)	Sequencing	Figure 2
JH19‐**ARF8**‐67	Line 15	(−2, −2)	PAM2(360 bp)	Sequencing	
JH19‐**ARF8**‐72	Line 1	(0, −2)	PAM2(360 bp)	Sequencing	
JH19‐**ARF8**‐100	Line 103	(−2, −2)	PAM2(360 bp)	Sequencing	Figure 2
JH19‐**ARF8**‐146	Line 19	(−2, −11 + 1S)	PAM2(360 bp)	Sequencing	Figure 2
JH19‐**ARF8**‐147	Line 19	(−2, −2)	PAM2(360 bp)	Sequencing	Figure 2
JH19‐**ARF8**‐149	Line 13	NA	PAM2(360 bp)	Sequencing	Large fragment deletion
JH19‐**ARF8**‐154	Line 1	NA	PAM1(340 bp)	Sequencing	Mismatch starts 140 bp upstream of PAM1
JH19‐**ARF8**‐105	Line 13	NA	PAM1(340 bp)	Sequencing	Mismatch starts 150 bp upstream of PAM1
JH19‐**ARF8**‐156	Line 7	(−2, −2)	PAM2(360 bp)	Sequencing	
JH19‐**ARF8**‐157	Line 7	(−2, −2)	PAM2(360 bp)	Sequencing	
JH19‐**ARF8**‐116	Line 29	NA	PAM2(360 bp)	Sequencing	
JH19‐**ARF8**‐391	Line 23	(0, −1 + 1S)	PAM2(360 bp)	Sequencing	1 bp deletion and 1 bp substitution

a − sign indicates deletion, 0 indicates no change. −1a, −1b refer to different 1 bp deletion alleles. 1S stands for single base substitution. NA: sequencing results are too complex to resolve, usually due to genetic mosaics and multiple alleles.

Among the twenty T0 transgenic plants harbouring the JH19‐FveARF8 construct (Figure 2b), fifteen (75%) showed mutations (Table 1; Figure 2d). Some plants such as plant #5 (0, −2) harboured heterozygous mutations (Figure 2d). Other plants were homozygous, such as plants #100 and #147 (−2, −2). Still others were biallelic such as plant #4 (−2, −3). In some cases, two or more plants were derived from the same transgenic line (same callus) but appeared to harbour different mutations such as plants #4 and #5 (Table 1; Figure 2d), indicating that Cas9‐mediated editing occurred after separate shoots were formed from the same callus. Interestingly, all mutations in *FveARF8* occurred at the PAM2 (TGG) site (Table 1).

In summary, most of the CRISPR‐induced mutations caused frameshifts near the N‐terminus of corresponding proteins, which are noted in Figure S5B,D.

### Inheritance and stability of CRISPR‐induced mutations in the T1 generation

**Figure 3:**
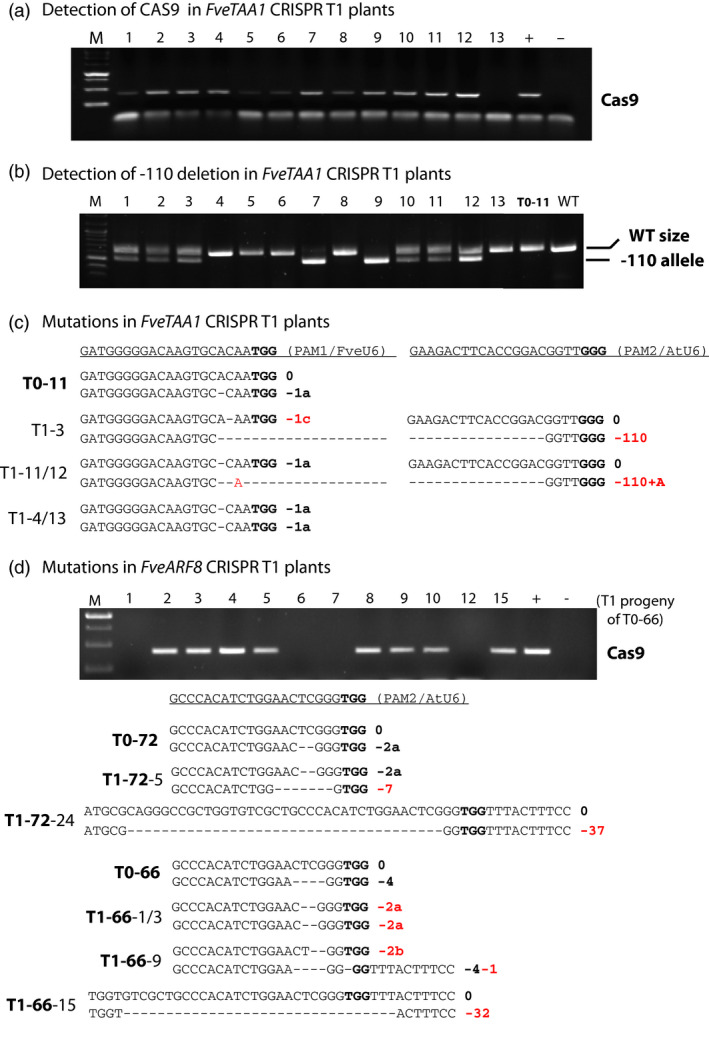
Genome editing continues at a high frequency in the T1 generation. (a) PCR gel image illustrating the presence of *Cas9* transgene in 12 of the 13 *JH19‐FveTAA1* T1 plants. All 13 are the self‐progeny of T0 plant #11 (T0‐11). + and – are respective positive and negative PCR controls. (b) PCR gel showing the presence of a −110 bp deletion in some of the *JH19‐FveTAA1* T1 plants. Plant 7 and 9 are homozygous for this deletion. T0‐11 (the T0 generation parent plant) and WT nontransgenic plant are shown as negative controls. (c) Illustration of mutations detected in *JH19‐FveTAA1* T1 plants. T0‐11 is the genotype of the T0 parent plant. Newly induced mutations (absent from T0‐11) are marked red including two large fragment deletions between the two PAM sites in plants T1‐3 and T1‐11 and T1‐12. (d) Transmission of the *JH19‐FveARF8* transgene (gel image) and mutations detected in the T1 plants derived from *JH19‐FveARF8* T0 plant #66 (T0‐66) or #72 (T0‐72). Newly induced mutations are marked in red.

The T0 transgenic plant #11 harbours JH19‐FveTAA1 and is heterozygous for a 1 bp deletion (0, −1a) (Figure 2c). To test for germ‐line transmission of the CRISPR‐induced mutation, seeds from self‐fertilized plant #11 were germinated and 13 T1 progeny were analysed by Sanger sequencing. Transmission of the initial mutation to the T1 progeny was observed. Specifically, two of the 13 plants were homozygous for the initial mutation (Table 2; Figure 3c). Interestingly, more than half (10 of 13) of the T1 plants carried new mutation(s); eight plants possessed an 110 bp fragment deletion between the PAM1 and PAM2 sites (Table 2; Figure 3b). All T1 plants with new mutations also harboured the *Cas9* transgene (Table 2; Figure 3a). The data suggested that T1 generation transgenic plants continued to generate new mutations at a high efficiency (10 of 12 or 83%). Interestingly, T1 plant #13, which was homozygous for the initial −1a mutation (Figure 3c), had lost the Cas9 transgene and is thus stabilized in its genotype (Figure 3a). This plant is both transgene‐free and homozygous for the desirable mutation (Table 2).

**Table 2:** Summary of genotyping results in T1 plants

Gene name and plant no.	New mutation	Genotype	PAM site	Cas9	Notes
JH19‐**TAA1**‐11‐1	NO	(−1a, −110)	PAM1&PAM2	+	Three sequencing peaks suggesting mosaic; Figure 3b
JH19‐**TAA1**‐11‐2	NO	(−1a, −110)	PAM1&PAM2	+	Figure 3b
JH19‐**TAA1**‐11‐3	YES	(−1c, −110)	PAM1&PAM2	+	Figure 3b,c
JH19‐**TAA1**‐11‐4	NO	(−1a, −1a)	PAM1	+	
JH19‐**TAA1**‐11‐5	YES	(0, −1a)	PAM1	+	
JH19‐**TAA1**‐11‐6	NO	(0, 0)	NA	+	
JH19‐**TAA1**‐11‐7	YES	(−110, −110)	PAM1&PAM2	+	Figure 3b
JH19‐**TAA1**‐11‐8	YES	NA	PAM1	+	Multiple peaks
JH19‐**TAA1**‐11‐9	YES	(−110, −110)	PAM1&PAM2	+	Figure 3b
JH19‐**TAA1**‐11‐10	YES	(−1a, −110)	PAM1&PAM2	+	Multiple peaks (mosaic); Figure 3b
JH19‐**TAA1**‐11‐11	YES	(−1a, −110/+1)	PAM1&PAM2	+	110 bp deletion between PAM1 and PAM2 plus +1 (Figure 3b,c)
JH19‐**TAA1**‐11‐12	YES	(−1a, −110/+1)	PAM1&PAM2	+	Same as JH19‐**TAA1**‐11‐11
JH19‐**TAA1**‐11‐13	NO	(−1a, −1a)	PAM1	−	
JH19‐**ARF8**‐72‐1	NO	(−2a, −2a)	PAM2	−	Figure 4b
JH19‐**ARF8**‐72‐2	YES	NA	PAM2	−	Multiple peaks
JH19‐**ARF8**‐72‐3	NO	(−2a, −2a)	PAM2	+	Figure 4b
JH19‐**ARF8**‐72‐4	NO	(0, −2a)	PAM2	+	
JH19‐**ARF8**‐72‐5	YES	(−2a, −7)	PAM2	+	Figures 3d and 4b
JH19‐**ARF8**‐72‐6	NO	(0, −2a)	PAM2	−	
JH19‐**ARF8**‐72‐7	NO	(0, −2a)	PAM2	+	
JH19‐**ARF8**‐72‐11	NO	(0, −2a)	PAM2	−	
JH19‐**ARF8**‐72‐12	NO	(0, −2a)	PAM2	‐	
JH19‐**ARF8**‐72‐24	YES	(0, −37)	PAM2	+	Figure 3d
JH19‐**ARF8**‐66‐1	NO	(−2a, −2a)	PAM2	−	Figures 3d and 4c
JH19‐**ARF8**‐66‐2	YES	(0, −2a)	PAM2	+	
JH19‐**ARF8**‐66‐3	NO	(−2a, −2a)	PAM2	+	Figures 3d and 4c
JH19‐**ARF8**‐66‐4	NO	(0, −2a)	PAM2	+	
JH19‐**ARF8**‐66‐6	NO	(0, −2a)	PAM2	−	
JH19‐**ARF8**‐66‐7	NO	(0, 0)	PAM2	−	
JH19‐**ARF8**‐66‐8	NO	(0, −2a)	PAM2	+	
JH19‐**ARF8**‐66‐9	YES	(−2b, −5)	PAM2	+	−5 = ‘−4 and −1’ (Figure 3d); Figure 4c
JH19‐**ARF8**‐66‐10	YES	(0, −2 + 1S)	PAM2	+	
JH19‐**ARF8**‐66‐15	YES	(0, −32)	PAM2	+	Figure 3d

NA: multiple peaks that are hard to resolve, likely caused by multiple mutations in different cells or due to polyploidy. −1a, −1b and −1c refer to different alleles of *TAA1*. −2a and −2b refer to different alleles of *ARF8*.

For *JH19‐FveARF8* transgenic plants, T1 progeny of two different T0 plants was analysed; they are #72 (0, −2) (transgenic line 1) and #66 (0, −4) (transgenic line 15) (Table 1; Figure 2d). The T1 progeny showed segregation of the mutation induced in T0 generation as well as new mutations induced in the T1 generation (Table 2; Figure 3d). Among the T1 progeny of plant #72, plants heterozygous and homozygous for the −2a deletion were identified (Table 2). Interestingly, while plant #66 was found heterozygous for a −4 deletion, most of its T1 progeny were heterozygous or homozygous for the −2a mutation (Table 2; Figure 3d), suggesting that the original #66 plant could be a genetic mosaic containing −4 and −2a mutation in different cells. Alternatively, the −2a mutation could have been generated in the T1 generation. Relatively large deletions (−32 and −37) were also found in *ARF8* in T1 plants (Figure 3d).

### *arf8* homozygous mutant seedlings showed faster growth and larger size

**Figure 4:**
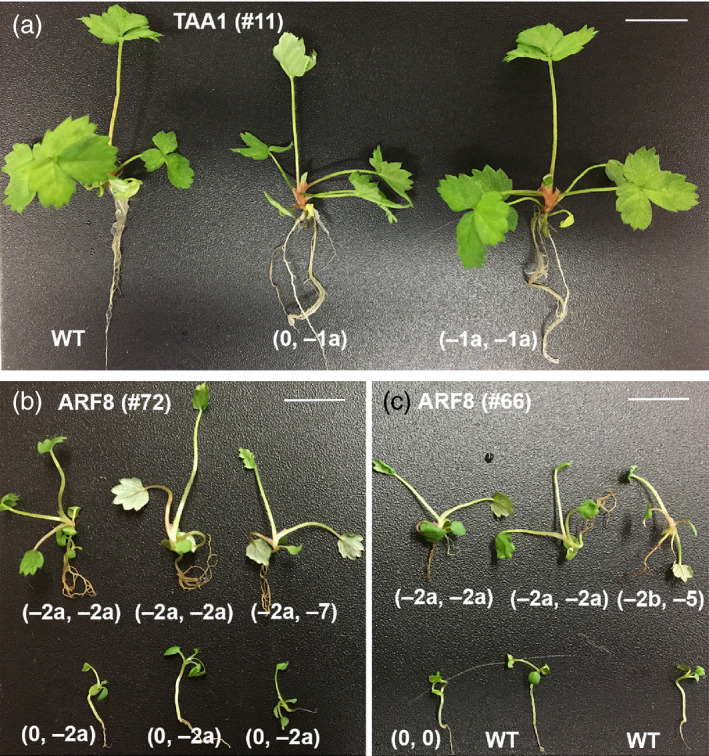
*arf8* mutants showed faster seedling growth. (a) Young *JH19‐FveTAA1* T1 plants germinated and grown in the same §‐strength MS medium at the same time. The three young plants are siblings derived from T0 plant #11, but differ in genotype (indicated beneath each plant). All three young plants look similar, suggesting that the homozygous mutation (far right) did not cause any obvious phenotype. (b) Six *JH19‐FveARF8* T1 seedlings derived from the same T0 plant #72. Homozygous or biallelic mutant seedlings (top row) all appear larger in size than the seedlings heterozygous for the *afr8* mutation (bottom row). All seedlings shown were germinated at the same time and grown in the same §‐strength MS medium. (c) *JH19‐FveARF8* T1 seedlings derived from a different T0 mother plant #66. Seedlings harbouring homozygous or biallelic mutations (top row) all appear larger in size than the seedlings that are wild type in genotype (bottom row). WT: YW5AF7

Auxin is an important phytohormone for plant growth and development; thus, we hypothesized that some of the homozygous knockout mutants might show abnormal phenotypes. As *TAA1* encodes an enzyme catalysing the key step in auxin biosynthesis, one would expect a reduction in plant height if auxin biosynthesis is adversely affected. Surprisingly, the young plants derived from T0 mother plant (T0‐11) all looked similar to one another (Figure 4a) despite that some young plants were homozygous for −1a deletion in *FveTAA1*. Examination of RNA‐seq data revealed that *FveTAA1* was only expressed at late‐stage anther (Figure S5E; Hawkins *et al*., 2017; Hollender *et al*., 2014). Therefore, *TAA1* may not play a role in seedling development. In contrast, *arf8* homozygous mutants from two different lines showed faster seedling growth compared to wild type (Figure 4b,c), even when the seedlings were germinated at the same time and grown in the same culture vessel.

### Examination of an off‐target site in T0 JH19‐FveARF8 plants

A potential off‐target site of *FveARF8*, Fvb1:18656977‐18656987, was identified based on CRISPRdirect (https://crispr.dbcls.jp/) (Naito *et al*., 2015). This off‐target sequence has 12 nucleotides matching the 20‐nucleotide seed sequence of *FveARF8* as well as three nucleotides matching the TGG PAM site (Table S1). Sequencing of PCR fragment flanking this off‐target site from five T0 plants and 10 T1 plants (still harbouring the JH19‐FveARF8 CRISPR construct) revealed an absence of any mutation at the off‐target site (Table S1).

## Discussion

Our data indicate that the CRISPR vectors developed here induce mutations at a high efficiency *in planta* ranging from 49% to 75% in the wild strawberry (YW5AF7). While this level of efficiency is based on two constructs targeting two genes, analysis of additional constructs targeting additional genes is showing similar range of mutational efficiency (Table S3). In this study, two genes with functions in auxin biosynthesis and signalling, *FveTAA1* and *FveARF8*, were targeted with two different target sites per gene. In total, 55 transgenic plants were tested, and 32 showed heterozygous, homozygous or biallelic mutations. Quite often, the mutational event occurred at only one of the two PAM sites per gene, suggesting that the dual sgRNA cassette increases the chance of at least one mutation in the targeted gene. Furthermore, successful editing by sgRNAs driven by either the AtU6‐26 promoter or the FveU6‐2 promoter indicates that both FveU6‐2 and AtU6‐26 promoters can work in strawberry and that sgRNA design and the promoter types may both contribute to the efficiency of CRISPR‐mediated genomic editing. Interestingly, all CRISPR‐induced mutations at *ARF8* occur at the PAM2 site where the sgRNA is driven by the *AtU6* promoter. The lack of mutation at the PAM1 site could be due to nonoptimal sgRNA design at the PAM1 site.

For the binary vector harbouring the Cas9 gene, constitutive promoters and tissue‐specific promoters were previously shown to induce Cas9 expression in higher plants, including the 35S promoter (Nekrasov *et al*., 2013; Shan *et al*., 2013; Xing *et al*., 2014), Ubiquitin (UBQ) promoter (Mao *et al*., 2013; Xing *et al*., 2014; Zhang *et al*., 2014), *YAO* promoter (Yan *et al*., 2015) and *ECL* promoter (Wang *et al*., 2015b). Here, we show that the *AtUBQ10* promoter is capable of driving *Arabidopsis/*maize codon‐optimized Cas9 gene expression and function in *F. vesca*. While we have not tested *in planta* the other binary vectors JH17 and JH16, they offer additional versatility in paring with specific sgRNAs or when they are applied to different transformation methods.

The JH4‐JH19 system reported here offered several advantages. First, one of the sgRNAs is driven by the strawberry U6 promoter (FveU6‐2), which is highly efficient for driving sgRNA expression and sometimes more efficient than the *Arabidopsis* promoter. Having both FveU6 and AtU6 promoters in a single vector maximizes the chance of successful CRISPR editing. Second, the gateway‐based two‐step cloning provides flexibility in constructing single, double, quadruple or higher order sgRNAs in pENTR vectors (Figure S1). Higher order sgRNA assembly can be easily achieved by SalI–XhoI enzyme digestion of one JH4‐2 sgRNA and ligation into a second JH4‐2sgRNA at the XhoI site. The process can be repeated to create higher order stacking. Third, the JH1 and JH4 pENTR vectors can be recombined with different destination vectors that contain the Cas9 gene. We have constructed three versions of such destination vectors including JH19 (*pUBQ::Cas9*), JH17 (*pYAO::Cas9*) and JH16 (*pECL::Cas9*) that can be used when applying different transformation methods. Fourth, the 35S::NLS‐3xGFP marker gene in the JH19 backbone offers the advantage of visual screening of positive transgenic callus, shoots and seedlings (Figure S2) as well as enabling the screen of loss of the Cas9 transgene for transgene‐free applications. Finally, our analysis of two target genes (*FveARF8* and *FveTAA1*) showed that the JH4‐JH19 system offers highly efficient genome editing in both T0 and T1 generations.

It is very encouraging that next‐generation (T1) JH19‐FveTAA1 and JH19‐FveARF8 plants continued genome editing with a high efficiency (even higher than the T0 generation for JH19‐*FveTAA1*). New types of mutations including large fragment deletions between the two PAM sites were generated in *FveTAA1* (Table 2; Figure 3b). Therefore, we can continuously screen for and obtain additional mutations in the following generations. This feature is especially attractive for CRISPR editing of cultivated strawberry, where continuous propagation of the transgenic plants containing the CRISPR construct may enable eventual editing of all eight alleles in the octoploid genome. Given the allopolyploid nature of the cultivated strawberry, the ability to stack multiple sgRNAs in the same construct each targeting a different allele may also facilitate genome editing in the cultivated strawberry.

*ARF8* in *Arabidopsis* was found to be a repressor of auxin signalling (Varaud *et al*., 2011). If it plays a similar role in strawberry, loss of *ARF8* function may lead to constitutive auxin responses.

Examination of RNA‐seq data of diploid strawberry showed that the *FveARF8* gene is expressed at a high level in floral organs and fruit, and it is also moderately expressed in seedlings (Figure S5F; Hawkins *et al*., 2017). This broad expression pattern of *FveARF8* suggests that *FveARF8* may repress auxin signalling in many tissues and developmental stages. Therefore, it is not surprising that *arf8* homozygous mutations cause a mutant seedling phenotype. Specifically, *arf8* mutant seedlings showed faster growth (Figure 4b,c). Among the T1 generation JH19‐FveARF8 plants, plant #72‐1 and plant #66‐1 have lost the transgene (based on PCR) and yet are homozygous for the −2 bp mutation (Table 2). Thus, they are valuable resources for further researches into the function of auxin during strawberry vegetative and reproductive development. Our work reported here demonstrates the exciting potential of CRISPR/Cas9 genome editing in improving traits of economically important *Rosaceae* fruit crops.

## Experimental procedures

### Plant materials, reagents and gene name assignment

The *Fragaria vesca* cultivar Yellow Wonder 5AF7 (YW5AF7) was used in this study. Plants were grown in a growth chamber at a temperature of 25 °C under 16‐h light and 22 °C in the dark, in a relative humidity of 50%. All the chemical reagents were purchased from VWR, Sigma‐Aldrich or Fisher. The restriction enzymes were from NEB. MS medium was purchased from RPI Company.

*FveTAA1* (gene03586) and *FveARF8* (gene31631) genes were selected based on their homology to the *Arabidopsis* and rice TAA1 and ARF8 protein sequence, respectively. Names were assigned based on BLAST and neighbour‐joining phylogenetic trees (Kang *et al*., 2013).

### sgRNA cassette design for Gateway pENTR vectors

Five U6 promoters from *Fragaria vesca* were identified by BLAST based on *Arabidopsis* U6‐26 and U6‐1 (Figure S6A). The conserved PSE (proximal sequence element: TGACGTAGGTYTYTCTCACCAGTCA) as well as the TATA box help indicate the transcription initiation site (Jensen *et al*., 1998). A double sgRNA cassette (Figure S6B) was designed and synthesized by Life Technology (Carlsbad, CA). The two sgRNAs are, respectively, driven by FveU6‐2 (scf0513178:1396765..1398764) and FveU6‐3 promoters (LG5:21684397..21686396) (Figure S6B). This synthesized fragment was inserted into the pENTR2b vector (Invitrogen, Carlsbad, CA) at SalI and EcoRI sites to yield JH2 vector. The two seed RNAs can be conveniently inserted into the type II restriction enzyme sites, BtgZ1 and BsaI, respectively.

However, the BtgZ1 enzyme site was found inefficient in cloning and was removed by SpeI and EcoR1 enzyme digestion and then replaced with an *Arabidopsis* U6 promoter‐driven sgRNA cassette (AtU6‐26 promoter/Linker/sgRNA scaffold/AtU6‐26 terminator) derived from pCAMBIA Cas9 + sgRNA (Jiang *et al*., 2013). This led to the dual sgRNA entry vector JH4 (Figure 1b). For this dual sgRNA entry vector, one seed RNA is inserted at the BsaI site driven by the FveU6‐2 promoter, and a second seed RNA is inserted into the JH4 vector via Q5 site‐directed mutagenesis kit (NEB, Ipswich, MA) and driven by the AtU6‐26 promoter.

For single sgRNA cassette JH1, the FveU6‐2 promoter‐sgRNA‐U6‐2 terminator fragment from JH2 was cut and released with EcoRI and SpeI and then inserted into pENTR2b at the EcoRI and XbaI sites to yield JH1 (Figure 1a). To insert the target seed sequence, 5′‐gctc G[19 bp target sequence]‐3′ is annealed to a reverse‐strand oligo (5′‐aaac G[19 bp in reverse complement]‐3′) to create the double‐stranded target sequence, which is inserted into JH1 at the Bsa1 sites (Figure 1a).

### Gateway destination vector construction

The destination vector JH12, a hygromycin‐resistant binary vector, was constructed to contain a Cas9 cassette amplified from pMOA33‐UBQ Cas9 OCS (Peterson *et al*., 2016). In this Cas9 cassette, a maize‐optimized *Cas9* gene and *Arabidopsis‐*optimized *Cas9* gene were synthesized with a haemagglutinin (HA) tag at the C‐terminus and fused to the N7 localization tag. The TMV omega translational enhancer was inserted between the *Arabidopsis* UBQ10 promoter and the Cas9 coding sequence, which ends with OCS terminator. This entire Cas9 cassette (from promoter to terminator) was inserted into pMDC99 (Curtis and Grossniklaus, 2003) at HindIII and KpnI sites to produce JH12 (Figure 1c).

The destination vector JH19 (Figure 1d) is similar to JH12 except that a 35S::NSL‐3XGFP cassette is inserted between KpnI and AscI restriction sites of JH12. Specifically, the 35S promoter was amplified from pGblog by primers 35S‐P‐F and 35S‐P‐R (containing KpnI and EcoRI sites, respectively). The NLS‐3XGFP fragment was cut from pGreenII‐NLS‐3XGFP‐GW (Zheng *et al*., 2011) by EcoRI and AscI sites. Then, the 35S promoter fragment and the NLS‐3XGFP fragment were inserted into JH12 at KpnI and AscI sites by three‐fragment ligation to yield JH19.

For the destination vector JH16 that contains egg‐cell‐specific *FveECL1* (gene02682) promoter‐driven *Cas9*, the attR1‐ccdb‐CMR‐attR2 (gateway cassette) fragment was cut from pMDC99 by HindIII and SpeI. This 1807 bp fragment was inserted into *PHEE401E* (Wang *et al*., 2015b) at HindIII and SpeI sites, resulting in *PHEE401E‐GW* (*JH15*). The *FveECL1* promoter was amplified by Q5^®^ High‐Fidelity DNA Polymerase (NEB) using primers ECL1‐P‐F1 and ECL1‐P‐R1 and substituted the EC1.2en/EC1.1p promoter in JH15 by SpeI and XbaI, which resulted in JH16.

For destination vector JH17 containing the *FveYAO* promoter‐driven *Cas9*, the *FveYAO* (gene 28947) promoter was amplified using primers FvYAO‐F1 and FvYAO‐R4 and substituted the EC1.2en/EC1.1p promoters in JH15 by SpeI and HpaI, which led to JH17.

### CRISPR/Cas9 editing of *ARF8* and *TAA1*

Two target sites of *FveARF8* in wild strawberry were determined by CRISPRdirect (https://crispr.dbcls.jp/) (Naito *et al*., 2015), and the potency score was measured by SSC (http://crispr.dfci.harvard.edu/SSC/) (Xu *et al*., 2015). Two guide RNA sequences were inserted into entry vector JH4 by BsaI digestion/ligation and Q5 site‐directed mutagenesis kit (NEB), respectively, through two primer pairs (Fv ARF8‐F/Fv ARF8‐R and Fv ARF8‐F3/Fv ARF8‐R3) (Table S2). The dual sgRNA cassette was recombined into JH19. *JH19‐FveTAA1* was constructed similarly using two different primer pairs (Fv TAA1‐F/Fv TAA1‐R and Fv TAA1‐F3/Fv TAA1‐R3) (Table S2).

### *Arabidopsis* protoplast transient assay and wild strawberry transformation

*Arabidopsis* protoplasts were isolated using the PEG method modified from Schapire and Lois (2016). The 35S::GFPm and JH12‐sgRNA constructs (10 µg each) were cotransfected into *Arabidopsis* protoplasts by the PEG method (Schapire and Lois, 2016) with JH12 harbouring a single sgRNA targeting the GFPm. The efficiency between *FveU6* promoter‐driven sgRNA and *AtU6* promoter‐driven sgRNA (both in the JH12 vector) was determined by counting GFP‐positive protoplasts under a ZEISS Axiovert 200 microscope.

*F. vesca* YW5AF7 was transformed using the method described previously (Caruana *et al*., 2018; Kang *et al*., 2013).

### Detection of genome editing events

T0 transgenic YW5AF7 plants were confirmed by checking for the presence of the transgene by PCR. *Cas9* was amplified with the primers ZaCas‐F2 and ZaCas‐R4. The DNA fragment containing the sgRNA region was amplified using primers FvU6‐P2‐F and FvARF8‐R (or Fv TAA1‐R for TAA1) (Table S2). To identify target site mutations, the genomic DNA fragment spanning the two PAM sites was amplified using primers ARF8‐F1‐1/ARF8‐R1‐1 and FvTAA1‐F1‐1/FvTAA1‐R1‐1, respectively (Table S2). To characterize T1 transgenic plants, the seeds from independent transgenic lines were germinated in §‐strength MS medium. Individual T1 plants were similarly characterized as the T0 plants described above.

Genomic DNA of transgenic plants was isolated following published protocol (Healey *et al*., 2014) with slight modifications. PCR was conducted using AccuStart II PCR ToughMix (Quanta Bio, Beverly, MA). For DNA sequencing, PCR fragments were purified by MP Geneclean iii kit (MP Biomedicals, Solon, OH) and sent for Sanger sequencing at Macrogen, USA (Maryland). Sequence results were analysed by DSDecode (http://dsdecode.scgene.com/home/) (Liu *et al*., 2015) to detect and resolve mutations. To further resolve ambiguity in some cases, the PCR fragments were cloned into pGEM^®^‐T cloning vector (Promega Co, Madison, WI), and plasmid DNAs from four to five colonies were sequenced.

### Detection of off‐target events

The potential off‐target sites for *FveARF8* were determined by CRISPRdirect (https://crispr.dbcls.jp/), and Fvb1:18656977‐18656987 off‐target site was chosen for analysis. The genomic DNA sequence across the off‐target site was amplified using primer pairs ARF8‐OT‐F2/ARF8‐OT‐R2 (Table S2) and then sequenced.

## Conflict of interest

The authors declare that there is no conflict of interest.

## Authors' contributions

JZ and ZL designed the experiments. JZ and GW performed the experiments. JZ and ZL analysed the data and wrote the manuscript.

## Supporting information


**Figure S1** Illustration of cloning strategies in moving single, double, or multiple sgRNAs from pENTR vectors (JH1 or JH4) into destination vectors.
**Figure S2** Nuclear localized 3XGFP driven by the 35S promoter serves as a marker for the transgene.
**Figure S3** Vector map of JH16 and JH17.
**Figure S4** Testing the efficiency of CRISPR/Cas9‐mediated genome editing in *Arabidopsis* protoplasts.
**Figure S5** Sequence and expression of *FveTAA1* and *FveARF8*.
**Figure S6** Identification and synthesis of FveU6 to drive sgRNA expression.

**Table S1** Summary of off‐target site sequencing results for T0 and T1 generation *JH19‐FveARF8* transgenic plants.
**Table S2** Primers used in this study.

**Table S3** Summary of genotyping results of additional T0 transgenic plants.
