# Supplementary material for: Efficient genome editing of wild strawberry genes, vector development and validation
Source: Plant Biotechnol J. 2018 Apr 24;16(11):1868–77. doi: 10.1111/pbi.12922 (PMC6181217; doi:10.1111/pbi.12922)
Supplement: Supplementary file 1 — Figure S1 Illustration of cloning strategies in moving single, double, or multiple sgRNAs from pENTR vectors (JH1 or JH4) into destination vectors. Figure S2 Nuclear localized 3XGFP driven by the 35S promoter serves as a marker for the transgene. Figure S3 Vector map of JH16 and JH17. Figure S4 Testing the efficiency of CRISPR/Cas9‐mediated genome editing in Arabidopsis protoplasts. Figure S5 Sequence and expression of FveTAA1 and FveARF8. Figure S6 Identification and synthesis of FveU6 to drive sgRNA expression. [file PBI-16-1868-s001.docx]

**Supporting Figures**

**Figure S1. Illustration of cloning strategies in moving single, double, or multiple sgRNAs from pENTR vectors (JH1 or JH4) into destination vectors.**

1. Sequential insertion of the first and second seed RNA into JH4.
2. Illustration of LR recombination reaction to move the sgRNA cassette into the destination vector of choice (JH12, JH19). JH16 and JH17 are also destination vectors for use, but are not shown in the diagram.
3. Illustration of stacking multiple target sgRNAs into the pENTR vector JH4. This can be easily achieved by SalI-XhoI enzyme digestion of one JH4-2sgRNA and ligation into a second JH4-2sgRNA at the XhoI site. This process can be repeated to create higher order stacking.

**
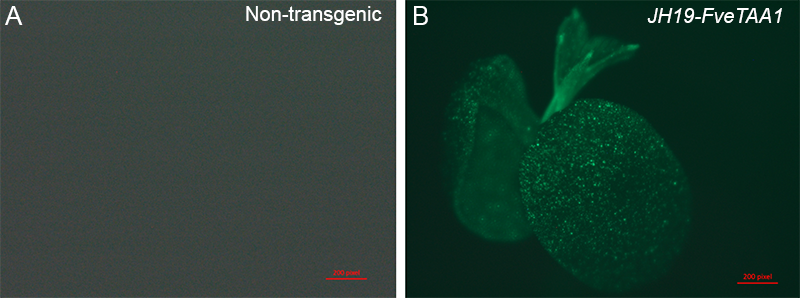
**

**Figure S2. Nuclear localized 3XGFP driven by the 35S promoter serves as a marker for the transgene**

1. A YW5AF7 seedling with no GFP signal.
2. A transgenic YW5AF7 seedling harboring the *JH19-FveTAA1* transgene. Nuclear GFP signals are seen in the cotyledons and hypocotyl.

**Figure S3. Vector map of JH16 and JH17**

1. JH16 plasmid map.
2. JH17 plasmid map.

**Figure S4. Testing the efficiency of CRISPR/Cas9-mediated genome editing in *Arabidopsis* protoplasts.** This is based on a frameshift mutation introduced into GFP (mGFP) that serves as a readout for CRISPR genome.

(A) Both FveU6 promoter driven sgRNA and AtU6 promoter driven sgRNA targeting the mGFP can correct the frameshift mutation and restore GFP activity. AtU6 driven sgRNA appears to have a higher efficiency than FveU6 driven sgRNA in restoring GFP. 1, 2, 3 refer to 35S::mGFP only, 35S::mGFP plus JH12-FveU6:sgRNA, and 35S::mGFP plus JH12-AtU6:sgRNA, respectively.

(B) Images showing GFP negative and positive protoplasts.

1. ***FveTAA1* (gene03586) sequence containing the target sites 1 and 2**

GGCACAAGAAGGTCTAGCGTTTCACCTGACACCGTCCTCAATCTCGAACAGTAAGTTCTTCGATCTTTAGCTTTCATCGTTCACCATCATCTTCTTCCAGAGATTCATTGAGCACTTCTAATATTATTGTTATATATACACACACGCCTCATCGATCTGCTGCATTGTTGAATAATGCAATGAGAGCCTGAATTGCAGTCAAGGAAAGAATGATTCAATTCCCAAAACTCAATTGGATCTTTCATATCATTCAATATGTGTGGTTCGAAATGTGTTTAAATGTTATACAACGACAAGCGTAACCCGTAGAATGATGTTTTATGGCAGGGGTGATCCAACTGTGTTTGAACCGTACTGGAAAAAGATGGGGGACAAGTGCACAA**TGG**TGATCTCCGGTGGCCAGTGGATGAGCTATGTGAGCGATTTCACCAACATCTGCTTCTTTTTGGAGCCAGAATTTGAGGATGCGGTTAGAAGACTTCACCGGACGGTT**GGG**AATGCGGTGGTGGACGGTCATTACATTGTGGTGGGGACAGGCTCCACGCAGCTCTACCAGGCGGCGCTGTATGCTCTCACTTCTCCTGGTGGGGCTGAGCCGATCAGTGTCGTGTCTGCCGCTCCTTACTACTCG

1. ***FveTAA1* (gene03586) amino acid sequence affected by the CRISPR mutations**

MCGTDHNPNPASPNKTPSANGNGTRRSSVSPDTVLNLEQGDPTVFEPYWKKMGDKC**TM**VISGGQWMSYVSDFTNICFFLEPEFEDAV**R**RLHRTVGNAVVDGHYIVVGTGSTQLYQAALYALTSPGGAEPISVVSAAPYYSFSVVVVLFMYRKTSATKLRALRVPDPLSYPDETDYLRSGLYRWAGDAYAFDKKGHGDYIEVVNTPNNPDGTNRKAVVKNRPDDQVQGKLIHDLAYYWPQYTPITAPVDEDIMYFTFSKSTGHAGSRIGWAIVKDKEVAKKMAKFVELSSLGVSKDSQHRAAKIMGVICDGYQNCKAKNPELFFEHCQNLMTERWERLREVVERIDIFSLPKYPKEYCLFSGEFNEPHPAFAWLKCKEDIDLEKFIRGCIKVQGRTGRKFGTDQNYLRISMLSKDEVFDHFLERLSTIKAISNGH-

Note: underlined amino acid sequence corresponds to PAM1 and PAM2 seed RNAs. -1a and -1b cause frameshift mutation at the PAM1 region starting at the highlighted **T** and **M** respectively. The 1S at PAM2 is a synonymous mutation (highlighted R).

1. ***FveARF8* (gene31631) sequence containing the target sites 1 and 2**

GATTCTCAGTCCACTTACTATTACAATCTCTTGAGAAGAAAAACAATTCCTCCCTTCAGTTTCTAATTCCTCCCAATTCGTGGAGTGTGAAAATAGAAAGTAGAATTTGGTTTCGACGTATGAGTTTAGTTGTGATAAGTAGTTGATAGGAAGGGAGGTGGATATCGGTATGAAGCTTTCCACATCAGGGTTCGGTCAGCAGGAAGGTAAACCCTATTGTTTGGTTGATTTTCGCATAGTAATCTAGTCCATTATCAACTGTATTTATTTATTGACAAGAAATGGAATGTCAGGAGGAGCTGAGAAGAAGTGCTTGAATTCGGAGCTGTGGCATGCATGCGC**AGG**GCCGCTGGTGTCGCTGCCCACATCTGGAACTCGGG**TGG**TTTACTTTCCTCAGGGCCACAGCGACCAGGTTGCGGCTACTACCAACAAACAAGTTGATGCTCACATACCAAACTACCCGAGTTTGCCTCCCCAGTTGATCTGTCAGCTCCACAATGTCACAATGCATGTGGGTTTTGCTTTGTCTTTTTTTATTCAGCTACATTTTGTCTTTTCATTTCTTGTAGAAGAAAGAACAAAAATGAGATAACCTGGAACTGATGTGATTTGGGTTTCAGGCAGATGTGGAGACGGATGA

1. ***FveARF8* (gene31631) amino acid sequence**

MSGGAEKKCLNSELWHACAGPLVSLPTS**GT**RVVYFPQGHSDQVAATTNKQVDAHIPNYPSLPPQLICQLHNVTMHADVETDEVYAQMTLQPLTPQEQKETFLPMELGVPSKQPTNYFCKTLTASDTSTHGGFSVPRRAAEKVFPPLDFSLQPPAQELIARDLHDVEWKFRHIFRGQPKRHLLTTGWSVFVSAKRLVAGDSVLFIWNEKNQLLLGIRRATRPQTVMPSSVLSSDSMHIGLLAAAAHASSTNSCFTVFYNPRASPSEFVIPLSKYIKAVFHTRVSVGMRFRMLFETEESSVRRYMGTITGISDLDPVRWPNSHWRSVKVGWDESTAGERQPRVSLWEIEPLTTFPMYPSLFPLRLKRPWHPGASSMHDNRDEAANLMWLRGATGEQGLQSMNFQAVGMFPWMQQRLDSTLMGNDPNQQYQAMLAAGLQNVGSGDQLRQHVMHFQQPLQYLQQPGSHNPLLQLQQQVIPQSVPHNMLQAQPQVSMENLPQHLLPPQFNNQTEEEPHQQQNTYHDALKVQSEQLHRSQQMNVPSPSFSRADYTDSSTKLSGSTNSRQNTLGSLCPEGSNSVLNRAGPAEQLPQQSWTPKFAYAQANAFANPMSFAPFNEKDNAVEQENCNSDSQNPTLFGVNIESSGLVFPTTVPNFATSSNDADMPMPLGDSGFQSSLYGCIQDSTELLHGAGQVDPPTPNCTFVKVYKSGSVGRSLDISRFSSYNQLREELAQMFGIEGKLEDCLRSGWQLVFVDREDDVLLLGDDPWESFVNNVWYIKILSPEDVHKMGDQAVDSFTPNAGQRVNSSANETQDHISGLPSLGSLEY-

Note: -1, -2, -4 all caused frameshift starting at the highlighted T. The -11 mutation caused frameshift starting at the highlighted G. The -3 mutation deletes T. All these mutations affect PAM2 region.

1. **Expression of *FveTAA1* across multiple tissues**


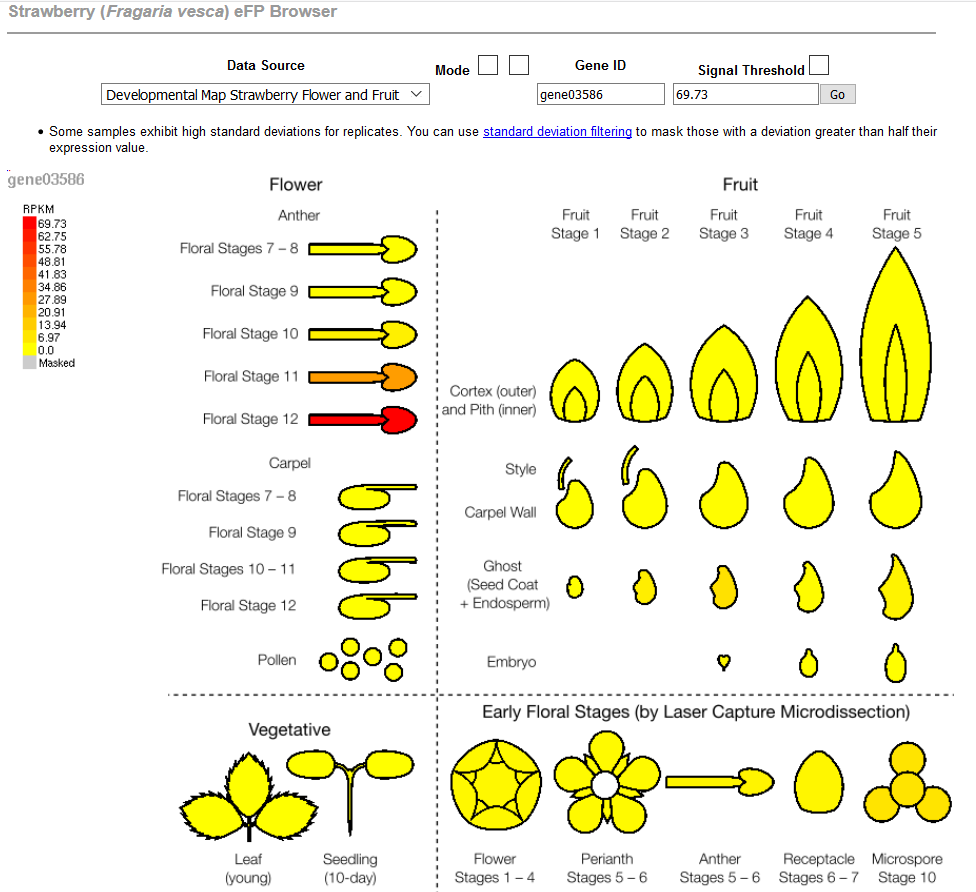


1. **Expression of *FveARF8* across multiple tissues**


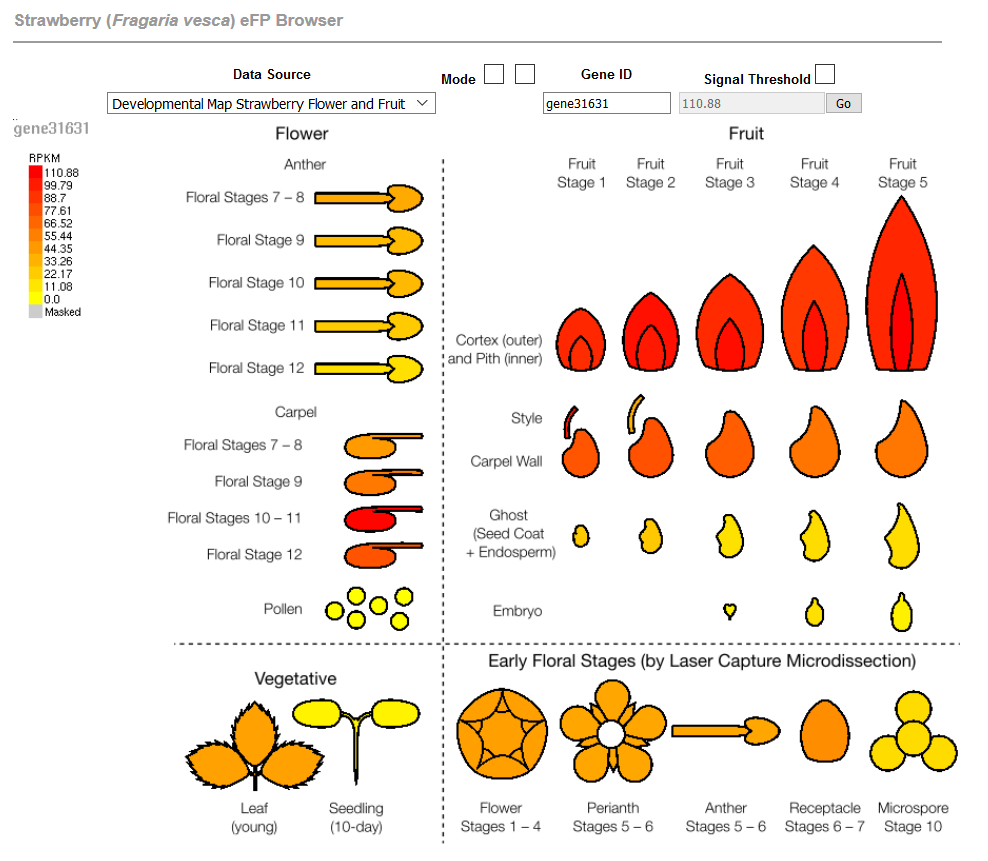


**Figure S5. Sequence and expression of *FveTAA1* and *FveARF8***

1. TAA1 (gene03586) sequence containing the target sites 1 and 2. The PAM1 (TGG) and PAM2 (GGG) are in bold. The seed RNAs are in red. Underlined are PCR primers.
2. Amino acids sequence of TAA1. Sequences corresponding to the PAM1 and PAM 2 seed RNAs are underlined. The impact of CRISPR-induced mutations on the protein product is noted.
3. ARF8 (gene31631) sequence containing the target sites 1 and 2. PAM1 (AGG) and PAM 2 (TGG) are in bold. The seed RNAs are in red. Underlined are PCR primers.
4. Amino acids sequence of ARF8. Sequence corresponding to the PAM 2 seed RNA is underlined. The impact of CRISPR-induced mutations on the protein product is noted.
5. eFP diagram showing *FveTAA1* expression in different *F. vesca* tissues.
6. eFP diagram showing *FveARF8* expression in different *F. vesca* tissues
7. **Five candidate FveU6 promoters and their alignment**


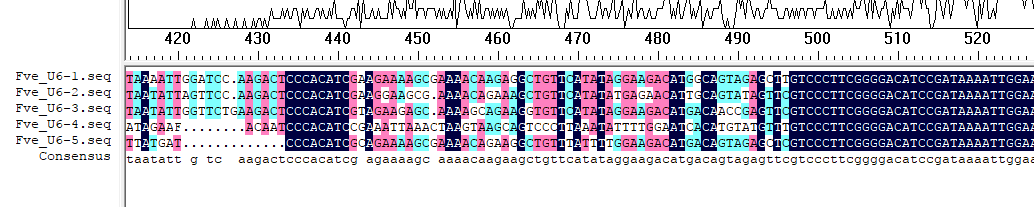


**PSE**

**TATA**

**U6**

**B. Synthesized sequence for the sgRNA pENTR cassette**

CACTATAGGGCGAATTGAAGGAAGGCCGTCAAGGCCGCATGTCGACAAGCTTCTTTTCTTGTTATAATAGTGTTGTTGT

ATTGTTCATTCAAAGACTGAACAAAAAAAGAAATTAGGTCAGTCAAATTGGTTTGTGTTTGTTGTAAAGTTTGTTGTAA

CACCAGTATATCCCGGTGGTCTAAGACGGGCAGTTTAGAGTCGTCCATTCTAACACGAATACATGATATTATCATGTGT

AAATCCTCAATGACTGCCTTCAAGCGCAACTTCAATTCATAAAAGTTTAATTTCAATTGTTAAACCTTCATTCTTCTAG

AATTGCACTTAAAGGAGGACACGTGAGGCGCACGGGCTTTTGTAATATTGGTTCTGAAGACTCCCACATCGTAGAAGAG

CAAAAGCAGAAGGTGTTCATATAGGAAGACATGACAACCGAGCTCGGCTAGGATCCATCGCAGTCAGCGATGAGTACAG

CAAGTTTTAGAGCTAGAAATAGCAAGTTAAAATAAGGCTAGTCCGTTATCAACTTGAAAAAGTGGCACCGAGTCGGTGC

TTTTTTTTTTTTGCCATTTTTCCCTCCCTCCTTTTTCGATTCAGGTAGTGTTTTCAGTTCTACTAGTAACGGCCGCCAG

TGTGCTGGAATTGCCCTTCTTCCAGAGTGGGATCTCGCTTTGAAATTAAAATGCGGGACTCCTTATTTCACTCACTTTC

AAGTCGTATTTCCACATCTCAACTGTACAATGTCTAAAACACAGTGTGTAGATCATTCATACAAAGTTCGTGTAAAGAT

TATTCAATGTATATTAACTTGGACAGGTGCCGTAAGCAGTCAAAATCTGTTCTGTTTTAAACCTTCATTCTTCTAGAAT

TGCACTTAAAGGGAGGACACGTGACGCGCACATGCTTTGGTAATATTAGTTCCAAGACTCCCACATCGAAGGAAGCGAA

AACAGAAAGCTGTTCATATATGAGAACATTGCAGTATAGCTCAGAGACCAAAGGAGGTCTCAGTTTTAGAGCTAGAAAT

AGCAAGTTAAAATAAGGCTAGTCCGTTATCAACTTGAAAAAGTGGCACCGAGTCGGTGCTTTTTTTTTTGGGGATTTTT

TCCCTCCTTGTTTGATTCCAGGTAGTGCTTCCAGATCTGGTGCTTTTCTCTCGAGGAATTCCTGGGCCTCATGGGCCTT

CCTTTCACTGCCCGCTTTCCAG

Yellow: sgRNA scaffold (Yang et al., NAR 2014)

Purple: Two inverted BtgZ1 recognition sites. They cut at the flanking sequence underlined.

Red: two inverted BsaI recognition sites. They cut at the flanking sequences underlined.

Underlined: overhang sticky ends produced upon restriction enzyme digestion. Seed RNA will be inserted between the paired sticky ends.

Green: U6-3 promoter

Blue: U6-2 promoter

**C. plasmid map containing the synthesized sequenc**e

**
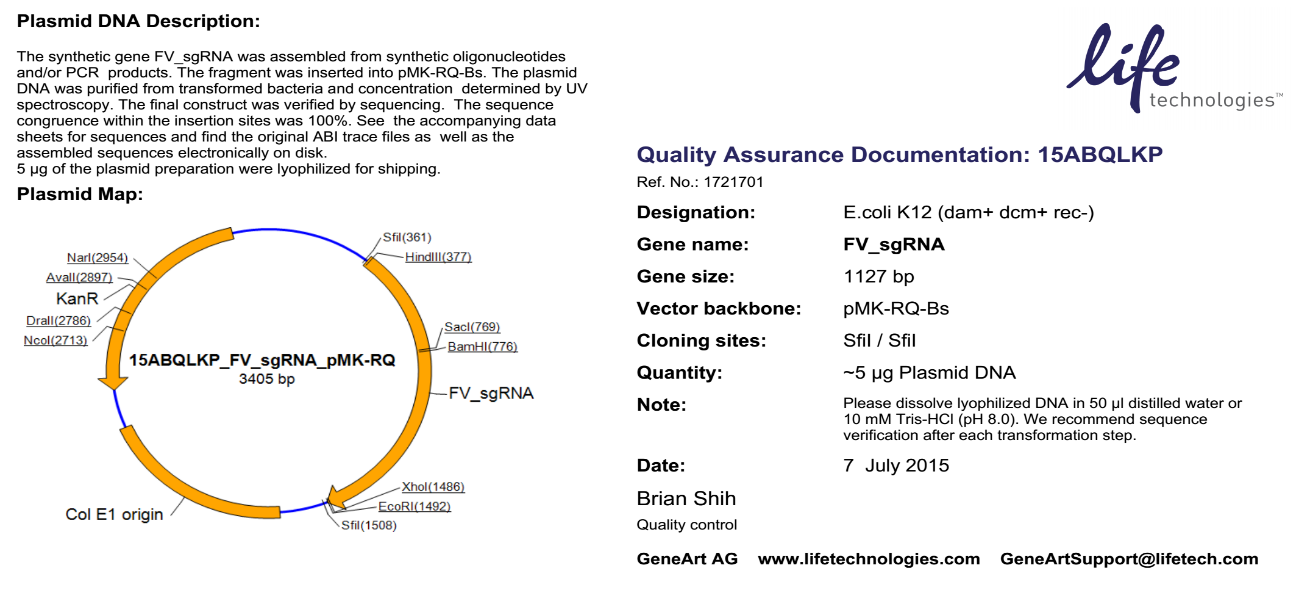
**

**Figure S6.** **Identification and synthesis of FveU6 to drive sgRNA expression**

(A). Alignment of five candidate FveU6 promoters. *Arabidopsis* U6-1 (accession X52527) and U6-26 promoters (accession X52528) sequences were blasted in GDR (https://www.rosaceae.org/) to identify these five candidate FveU6 promoters (scf0513173:315641..317640, scf0513178:1396765..1398764, LG5:21684397..21686396, scf0513160:642074..644073, LG5:21433753..21435752). The conserved Proximal Sequence Element (PSE) and the TATA box help identify the transcription initiation site. The promoter sequence as well as the terminator sequence was used in designing the cloning cassette shown in B.

(B). Sequence of the synthesized double sgRNA cassette. The two identical sgRNA scaffolds are highlighted in yellow and is based on the sgRNA from Jiang et al. (2013). Each sgRNA scaffold is driven by a FveU6 promoter highlighted in green (U6-3) or blue (U6-2). A pair of type II restriction enzyme recognition sites, BtgZ1 (purple) or BsaI (red), flanked by their cleavage sites (underlined) are placed between the FveU6 promoter and the sgRNA scaffold. 20bp seed RNA sequence will be inserted into the Btgz1 or BsaI site.

(C). Plasmid map from Life Technologies. The plasmid contains the synthesized sequence shown in (B).
