# Supplementary material for: Efficient genome editing of wild strawberry genes, vector development and validation
Source: Plant Biotechnol J. 2018 Apr 24;16(11):1868–77. doi: 10.1111/pbi.12922 (PMC6181217; doi:10.1111/pbi.12922)
Supplement: Supplementary file 2 — Table S1 Summary of off‐target site sequencing results for T0 and T1 generation JH19‐FveARF8 transgenic plants. Table S2 Primers used in this study. [file PBI-16-1868-s002.docx]

Table S1. Summary of off-target site sequencing results for T0 and T1 generation *JH19-FveARF8* transgenic plants.

| Plant Name | T0 or T1  (line No.) | ARF8 genotype at PAM 2 site | Off-target site 1  Sequencing result (no change)* |
| --- | --- | --- | --- |
| WT (YW5AF7) |  | GCCCACATCTGGAAATCGGGTGG | GTAGAGAGAAGAAAATCGGGTGG |
| JH19-FveARF8-4 | T0 (#102) | (-2, -3) | GTAGAGAGAAGAAAATCGGGTGG |
| JH19-FveARF8-5 | T0 (#102) | (0, -2) | GTAGAGAGAAGAAAATCGGGTGG |
| JH19-FveARF8-100 | T0 (#103) | (-2, -2) | GTAGAGAGAAGAAAATCGGGTGG |
| JH19-FveARF8-146 | T0 (#19) | (-2, -11+1S) | GTAGAGAGAAGAAAATCGGGTGG |
| JH19-FveARF8-147 | T0 (#19) | (-2, -2) | GTAGAGAGAAGAAAATCGGGTGG |

| JH19-FveARF8-66-1 | T1 (Line 15) | (-2a, -2a) | GTAGAGAGAAGAAAATCGGGTGG |
| --- | --- | --- | --- |
| JH19-FveARF8-66-3 | T1 (Line 15) | (-2a, -2a) | GTAGAGAGAAGAAAATCGGGTGG |
| JH19-FveARF8-66-4 | T1 (Line 15) | (0, -2a) | GTAGAGAGAAGAAAATCGGGTGG |
| JH19-FveARF8-66-7 | T1 (Line 15) | (0, 0) | GTAGAGAGAAGAAAATCGGGTGG |
| JH19-FveARF8-66-9 | T1 (Line 15) | (-2b, -5) | GTAGAGAGAAGAAAATCGGGTGG |
| JH19-FveARF8-72-1 | T1 (Line 1) | (-2a, -2a) | GTAGAGAGAAGAAAATCGGGTGG |
| JH19-FveARF8-72-3 | T1 (Line 1) | (-2a, -2a) | GTAGAGAGAAGAAAATCGGGTGG |
| JH19-FveARF8-72-4 | T1 (Line 1) | (0, -2a) | GTAGAGAGAAGAAAATCGGGTGG |
| JH19-FveARF8-72-5 | T1 (Line 1) | (-2a, -7) | GTAGAGAGAAGAAAATCGGGTGG |
| JH19-FveARF8-72-24 | T1 (Line 1) | (0, -37) | GTAGAGAGAAGAAAATCGGGTGG |

*Note: Red nucleotides are homologous between *ARF8* PAM site 2 and the off-target site

| **Table S2: Primers used in this study** | | |
| --- | --- | --- |
| Primer name | Primer sequence | Notes |
| ZaCas9-F | 5-cccAAGCTTatgcatatgagtctagctcaac-3 | PCR primer amplifying a 7.2 kb cassette of UBQ10-ZaCas9 –OCS (HindIII&KpnI) |
| ZaCas9-R | 5-ggGGTACCagatttaggtgacactatagaata-3 |  |
| ZaCas-F2 | 5-GGACTAGTatggacaaaa aatacagcattg-3 | PCR primer for screening transgenic plants, amplifying a 420bp ZaCas9 fragment |
| ZaCas-R4 | 5-cttgcgaagatggtagatcgtg-3 |  |
| PENTR2b-F | 5-GCGTTTCTACAAACTCTTCC-3 | PCR primer annealing to upstream of attL1 in PENTR2b for validating insertion of guide RNA |
| PENTR2b-R | 5-CATCAGAGATTTTGAGACACGG-3 | PCR primer annealing to downstream of attL2 in PENTR2b for validating insertion of guide RNA |
| Fv YAO-F1 | 5’-CCTTAATTAAACTAGTGTCTTGCTACCTCGGCAACA-3’ | For amplifying FveYAO promoter, containing PacI and SpeI enzyme sites |
| Fv YAO-R4 | 5’-cggttaacCGGAGTTGGCGCTGCTGGGT-3’ | For amplifying FveYAO promoter, containing HpaI enzyme site |
| ECL1-P-F1 | 5’-CCTTAATTAAAGACTAAGACTAGTTCTAGTG-3’ | For amplifying FveECL1 promoter, containing PacI/SpeI sites, |
| ECL1-P-R1 | 5’-GCTCTAGATTTTACCAATCAAAGATTACAACT-3’ | For amplifying FveECL1 promoter, containing XbaI enzyme site |
| Fv TAA1-F | 5’-gctcGATGGGGGACAAGTGCACAA-3’ | For inserting 1^st^ FveTAA1 seed RNA into JH4 (PAM1/FveU6) by BsaI digestion-ligation. |
| Fv TAA1-R | 5’-aaacTTGTGCACTTGTCCCCCATC-3’ |  |
| Fv ARF8-F | 5’-gctcGAGCTGTGGCATGCATGCGC-3’ | For inserting 1^st^ FveARF8 seed RNA into JH4 (PAM1/FveU6) by BsaI digestion-ligation. |
| Fv ARF8-R | 5’-aaacGCGCATGCATGCCACAGCTC-3’ |  |
| GFPm-F | 5'-gctcGCGCTTCAAGGTGCACATGG-3' | For inserting 1st GFP seed RNA into JH4 (or JH1) via BsaI digestion-ligation. |
| GFPm-R | 5'-aaacCCATGTGCACCTTGAAGCGC-3' |  |
| GFPm-Q5-F1 | 5'- gtgcacatggGTTTTAGAGCTAGAAATAGCAAG-3' | For inserting 2nd GFP seed RNA into JH4 (PAM2/AtU6) by Q5 site directed mutagenesis kit. |
| GFPm-Q5-R1 | 5'-cttgaagcgcAATCACTACTTCGACTCTAG-3' |  |
| ARF8-F1-1 | 5’-GATTCTCAGTCCACTTACT-3’ | PCR primer amplifying both ARF8 target sites for confirmation of JH19-ARF8 transgenic plants. |
| ARF8-R1-1 | 5’-TCATCCGTCTCCACATCTGC-3’ |  |
| Fv ARF8-F3 | 5’-AGTAGTGATT GCCCACATCTGGAACTCGGGGTTTTAGAGCTAGAAATAGCAAGTTAA-3’ | For inserting 2^nd^ FveARF8 guide RNA into JH4 (PAM2/AtU6) by Q5 site directed mutagenesis kit. |
| Fv ARF8-R3 | 5’-CTAGCTCTAAAACCCCGAGTTCCAGATGTGGGCAATCACTACTTCGACTCTAGCTGTATA-3’ |  |
| Fv TAA1-F3 | 5’AGTAGTGATTGAAGACTTCACCGGACGGTTGTTTTAGAGCTAGAAATAGCAAGTTAAAATAAG-3’ | For inserting 2^nd^ FveTAA1 seed RNA into JH4 (PAM2/AtU6) by Q5 site-directed mutagenesis kit. |
| Fv TAA1-R3 | 5’CTAGCTCTAAAACAACCGTCCGGTGAAGTCTTCAATCACTACTTCGACTCTAGCTGTATATAAAC-3’ |  |
| Fv TAA1-F1-1 | 5’-GGCACAAGAAGGTCTAGCGT-3’ | For confirmation of the JH19-TAA1 transgenic plants. The primer pair amplifes across both TAA1 target sites, yielding a 630bp fragment. |
| Fv TAA1-R1-1 | 5’-CGAGTAGTAAGGAGCGGCAG-3’ |  |
| FvU6-P2-F | 5'-CTTCCAGAGTGGGATCTCGC-3’ | Binding to 5' end of FvU6-2 promoter |
| AtU6-P-F1 | 5'-GGGTCGACGTAAAGCCTGTAG-3’ | Amplify the entire *Arabidopsis* U6 driven sgRNA cassette from the AtU6-promoter (containing a Sal1 site) to the AtU6-terminator (containing a SpeI site) from the pCAMBIA+Cas9+sgRNA vector (Jiang et al., 2013). The fragment was inserted into JH2 to generate the JH4 vector |
| AtU6-P-R1 | 5'-ggACTAGTCGATCTGGAAAATTTTGCAA-3' |  |
| 35s-P-F | 5'-ggGGTACCtcccacaaaaatctgagctt-3’ | For amplifying 35S-promoter from Pgblog, containing KpnI-EcoRI sites for driving 3XGFP in constructing JH19 |
| 35S-P-R | 5'-CGGAATTCagcgtgtcctctccaaatgaa-3' |  |
| NLS-F | 5'-CGGAATTCatggctccaaagaagaagag-3’ | For amplifying the NLS-3XGFP cassette (from NLS to the NOS Terminator) from pGreenII-NLS 3XGFP-GW (Takada and Jürgens, 2007). The primers contain EcoRI and AscI site respectively. This fragment and the 35S promoter fragment (see row above) were ligated into JH12 (via KpnI and AscI sites). |
| NosT | 5'-AGGCGCGCCgaacaaaagctggagctcca-3' |  |
| JH19-seq | 5'-gaggatccactagttctaga-3' | JH19 sequencing primer |
| ARF8-OT-F2 | 5'-ATACCAAGCTTGCCCGGAT-3' | ARF8 off-target site 2 examine primers |
| ARF8-OT-R2 | 5'-GAGTAAATAGTTTAATTGGTCC-3' | ARF8 off-target site 2 examine primers |
