# Supplementary material for: Efficient genome editing of wild strawberry genes, vector development and validation
Source: Plant Biotechnol J. 2018 Apr 24;16(11):1868–77. doi: 10.1111/pbi.12922 (PMC6181217; doi:10.1111/pbi.12922)
Supplement: Supplementary file 3 — Table S3 Summary of genotyping results of additional T0 transgenic plants. [file PBI-16-1868-s003.docx]

**Table S3.** **Summary of genotyping results in T0 transgenic plants containing additional CRISPR constructs**

| Construct name | Number of transgenic plants containing the CRISPR/Cas9 construct | Number of plants  with mutations in the target gene | Mutation rate |
| --- | --- | --- | --- |
| JH19-Gid1a | 8 | 3 | 37% |
| JH12-ARF8 | 5 | 4 | 80% |
